# The effectiveness and safety of Chuna manual therapy adjuvant to Western medicine in patients with chronic obstructive pulmonary disease: Study protocol for a randomized, single-blind, investigator-initiated, pilot trial

**DOI:** 10.1097/MD.0000000000027217

**Published:** 2021-09-24

**Authors:** Kwan-Il Kim, Beom-Joon Lee, Hee-Jae Jung

**Affiliations:** a Department of Clinical Korean Medicine, Graduate School, Kyung Hee University, Dongdaemun-gu, Seoul, Republic of Korea; b Division of Allergy, Immune and Respiratory System, Department of Internal Korean Medicine, Kyung Hee University, Dongdaemun-gu, Republic of Korea.

**Keywords:** add-on, chronic obstructive pulmonary disease, Chuna, efficacy, safety

## Abstract

**Background:**

In chronic obstructive pulmonary disease (COPD) management, greater emphasis has been placed on symptomatic improvement and enhanced quality of life in patients. Manual therapy among respiratory rehabilitation programs has received much attention recently, with the publication of numerous studies. In South Korea, a method known as Chuna Manual Therapy (CMT) has been applied in the management of COPD patients and in clinical practice, but the clinical basis for safety and effectiveness is yet to be established. Therefore, rigorously designed randomized controlled trials are required. We aimed to evaluate the feasibility of assessing the add-on effect and safety of CMT administered with standard Western medicine therapy for the treatment of COPD.

**Methods:**

This is a randomized, single-blind, single-center clinical pilot trial. Patients with COPD receiving standard drug therapy are randomly divided into an experimental group (n = 20) and a control group (n = 20) at a 1:1 ratio. The experimental group receives CMT adding to the standard medical therapy once a week for 8 weeks. The control group receives only the standard drug treatment. The trial is conducted with an outcome assessor and statistician blinding. The primary outcome is the 6-minute walk test. The secondary outcomes include the pulmonary function test, the Modified Medical Research Council, visual analog scale for dyspnea, COPD assessment test, quality of life using the St. George's respiratory questionnaire, EuroQOL five dimensions questionnaire, and Korean pattern identification questionnaire. Adverse events are also be evaluated.

**Conclusions:**

The results of this study will provide the feasibility of a large-scale clinical trial to establish high-quality clinical evidence of CMT for COPD.

**Trial registration:**

Korean Clinical Trial Registry (http://cris.nih.go.kr; registration number: KCT0006119).

## 1 Introduction

Chronic obstructive pulmonary disease (COPD) is a common respiratory disease characterized by persistent respiratory symptoms and airflow limitation.^[1]^ Immediate effect of manual therapy (MT) on respiratory functions and inspiratory muscle strength in patients with COPD. Chronic inflammation and obstruction of airflow in COPD reduce the interstitial elasticity, while the prolonged contraction of respiratory muscles leads to an increased ventilatory flow demand, placing a substantial burden on the respiratory muscles.^[2,3]^ The consequent pulmonary hyperinflammation or increased respiratory demand causes breathing distress, a change in breathing patterns, or a fall in motor ability. As a result, COPD patients experience limited and reduced physical activity, which may cause skeletal muscle wasting, sarcopenia, or cachexia.^[4]^ In recent studies, therefore, emphasis has been placed on nonpharmacological therapy, including respiration rehabilitation, for managing the underlying symptoms of COPD.^[1]^ In respiration rehabilitation, the main focus has been on exercise therapy, and recently, MT has received much attention. MT has been shown to indirectly improve motor ability and lung function in patients by increasing thoracic wall flexibility and chest exercise.^[5,6]^

In South Korea, Chuna manual therapy (CMT) has been used for the treatment of various diseases, including musculoskeletal conditions. CMT is performed by a Korean medicine doctor who uses his or her hands or other parts of the body and an assistive tool such as the Chuna table to provide effective stimuli to the patient's body for the treatment of structural or functional problems. While it may share similarities with other manual therapies performed in different countries, the Korean CMT has advanced as a unique technique that integrates the outstanding manual therapies of China, Japan, India, and the US based on the traditional Chuna technique with the Korean traditional meridian theory.^[7]^ CMT is generally applied in various treatments of the musculoskeletal system,^[8,9]^ but its effects have also been reported for headache, tinnitus, and digestive diseases.^[10–13]^ MT in COPD patients has been investigated with systematic reviews^[14–18]^; however, the clinical efficacy of CMT has not yet been studied. Thus, this study aimed to investigate the safety and effectiveness of CMT in COPD through a small-scale, exploratory clinical study for the combined treatment of CMT and standard drug therapy.

## 2 Methods

### 2.1 Objective and design

**Figure 1:**
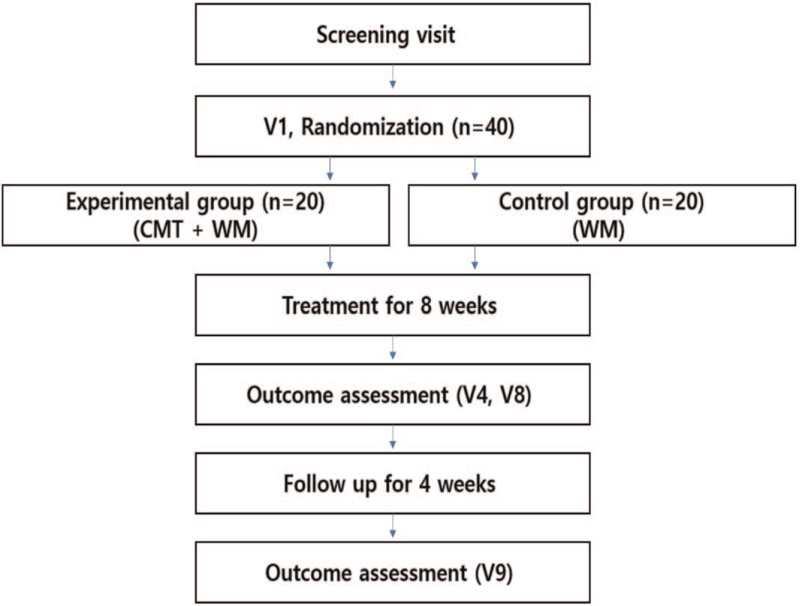
Flowchart of the study. CMT = Chuna manual therapy; WM = Western medicine.

The primary purpose of this study is to verify the effectiveness and safety of CMT in combination with Western medicine therapy for COPD. The patients receiving COPD treatments are randomized into two groups: the experimental group for a combination of CMT and Western medicine therapy and the control group for Western medicine therapy only, while the intervention is performed for 8 weeks, once a week. The study will be conducted as a prospective, single-center, single-blinded, exploratory clinical study, and based on the results, the sample size for future large-scale clinical trials will be determined. The feasibility of the study protocol will also be evaluated. A flow diagram of this study is shown in Figure 1.

### 2.2 Randomization, allocation, and blinding

The patients are randomized into two groups: the experimental group (standard Western medicine therapy + CMT) and the control group (standard Western medicine therapy), in a 1:1 ratio. For randomization, an independent statistician produced a random number table using Microsoft Windows SAS ver. 9.1.3 (SAS Institute Inc, NC, Cary) and allocates random numbers to the patients.

The random number table produced using the SAS and the allocation table for the test or the control group based on the random numbers are stored by the statistician until discontinuation or termination of the study, while the principal investigator does not request for the data.

The subjects in this study are given the screening number as they submit the signed written consent for participation in the clinical study after receiving an explanation regarding the study.

The subjects finally selected through the screening test are given the registration number, and based on the random number table stored by the statistician, each subject is allocated to the group in line with the subject registration number. This study aims to investigate CMT as an add-on to standard drug therapy, and it will be conducted as a single-blinded study with a control group receiving only standard drug therapy. Neither the subjects nor the Korean medicine doctor practitioners is blinded. The principal investigator and assessor who collecting the data are blinded to each group allocation.

### 2.3 Patients

#### 2.3.1 Inclusion criteria

1. Patients aged between 40 and 80 years
2. Patients who satisfy the COPD clinical diagnostic criteria (forced expiratory volume in one second [FEV1] / forced vital capacity [FVC] < 0.70 on the Spirometry)^[1]^ and receiving the standard drug therapy
3. Patients who voluntarily signs and submits the written consent for participation in the present clinical study

#### 2.3.2 Exclusion criteria

1. Individuals with comorbidity of severe respiratory disease other than COPD (cystic fibrosis, pneumonia, interstitial lung disease, lung cancer, etc)
2. Individuals with a history of alcohol or other substance abuse or dependency
3. Individuals showing a clinically significant disease or disorder of the liver, kidneys, cardiovascular system, respiratory system, endocrine system, and central nervous system, based on the results of the physicochemical test and clinical examination, and those with a history of malignant tumor or mental disorder (here, individuals without 5-year postoperative recurrence are allowed to participate)
4. Individuals showing a change of drug use within the past three months prior to the present study
5. Individuals who are unable to walk independently
6. Individuals who have received kinetic, physical, or MT related to respiratory rehabilitation within the past month
7. Individuals who are currently receiving an oxygen therapy
8. Individuals with symptoms that prevent the use of CMT
9. Individuals who are pregnant or planning a pregnancy
10. Individuals who are deemed unsuitable for the clinical study by the principal investigator

### 2.4 Intervention

The CMT is performed on the primary and accessory respiratory muscles. The CMT in this study is given to all subjects in the same way from the first to the tenth method, the details of which are as follows.

1. Cervical relaxation: The patient is in a supine position, and the therapist uses both hands to cover the patient's neck and turns the C1-7 spinous process and splenius muscle with pressure using the second and third fingers.
2. Occipitocervical junction relaxation: The patient's head is made to protrude from the bed, and the therapist uses the second, third and fourth fingers to push the occipitocervical junction with pressure.
3. Trapezius muscle relaxation: The therapist uses the thumb and the second finger to lift the trapezius muscle on both sides, and pushes the muscle with the thumb with pressure for relaxation.
4. Clavicle relaxation: The therapist uses the thumb to repeatedly rub the upper and lower sides of the clavicle for relaxation.
5. Pectoral major and latissimus dorsi muscle relaxation: The therapist stretches the hands and with pressure on the thumb pushes the entirety of the pectoral major muscle with pressure for relaxation. Next, using the thumb, the therapist pushes with pressure the insertion point of the latissimus dorsi muscle vertically below the armpit.
6. Rectus abdominal muscle relaxation (via upper limbs): The therapist holds both hands of the patient to help him or her stretch the arms upwards, then stretch the feet while breathing out (1 set). Next, the patient is guided to bring the feet back towards the body while breathing out (2 sets) → The two sets are repeated.
7. Quadratus lumborum and intercostal muscle relaxation (via upper limbs): In position 6, the therapist pulls the patient's arm to the left. Here, the patient is instructed to move their feet to the left (one set). Next, the therapist pulls the patient's arm to the right, while the patient moves the feet to the right (two sets) → The two sets are repeated.
8. Thoracic breathing relaxation (via pressure on the humeral head): The therapist places the palms on the patient's humeral head on both sides, and applies pressure as the patient breathes in and relaxes as the patient breathes out. This process is repeated.
9. Breathing relaxation (via pressure on pectoral major muscle): The therapist places the palms on the patient's pectoral major on both sides, and applies pressure as the patient breathes in and relaxes as the patient breathes out. This process is repeated.
10. Abdominal trapezius and thoracolumbar paraspinal muscle relaxation: The therapist places the palms on the patient's abdominal trapezius muscle and thoracolumbar paraspinal muscle, and applied pressure in a soft sliding motion from the area around C7, along with the rectus abdominis muscle, then relaxes toward the waist area for overall muscle relaxation.

The CMT is performed by a Korean medicine doctor with over 3 years of experience over 15 minutes.

### 2.5 Outcome measures

#### 2.5.1 Primary outcome

Primary outcome is the 6-minute walk test (6-MWT). The 6-MWT is a relatively simple and economic test that measures the distance the patient can walk on a flat, hard surface for 6 minutes. The test is commonly used to assess motor function in COPD patients,^[19]^ and it has been regarded as a crucial indicator of exercise tolerance in COPD patients across numerous studies.^[20–23]^

The clinical trial process in presented in Table 1.

**Table 1:**
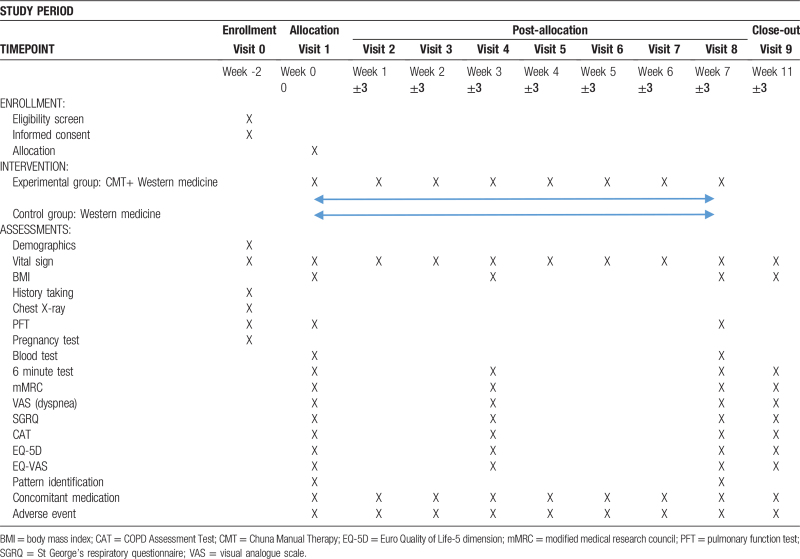
Trial schedule.

#### 2.5.2 Secondary outcomes

Secondary outcomes include the pulmonary function test, the Modified Medical Research Council,^[24]^ and the visual analog scale for measuring respiratory distress. The St. George's Respiratory Questionnaire,^[25]^ a COPD assessment test,^[26]^ and EuroQOL five dimensions questionnaire (EQ-5D),^[27]^ are used to measure the quality of life. To classify the idiopathic types of COPD and evaluate treatment efficacy, pattern identification is carried out.

### 2.6 Safety

Adverse events (AEs) refer to undesirable, unintended signs, symptoms, or diseases that occur during clinical trials and do not necessarily have causal relationships with the treatments used in the trial. The presence of AEs should be checked at every visit. The severity of AEs is measured using the Common Terminology Criteria for Adverse Events (CTCAE); Grade 1 (Mild): The normal daily activities (functions) are not prevented with only minimal discomfort and the subject can tolerate the symptoms; Grade 2 (Moderate): the normal daily activities (functions) of the subject are significantly inhibited; Grade 3 (Severe or medically significant): The normal daily activities (functions) of the subject are completely inhibited; Grade 4 (life-threatening consequences); Grade 5 (death related to AE). A serious adverse event (SAE) refers to one of the following AEs occurring in a clinical study participant during a clinical trial:

1. death or danger to life;
2. hospitalization or extension of hospital stay due to an AE;
3. permanent or significant failure or degradation of function;
4. development of fetal malformations or abnormalities; and
5. other medically important situations.

In the case of SAEs, investigators should report to the sponsor very quickly (usually within 24 hours).

### 2.7 Sample size

This study is a small-scale, prospective, preliminary clinical study to verify the feasibility and treatment effects of a large-scale clinical trial. Therefore, an accurate sample size must not be determined. Based on a previous study suggesting a minimum of 12 subjects per group for a pilot study in biomedical science,^[28]^ the number of subjects to be recruited and the minimum range for efficacy evaluation is set as 16, considering a 20% dropout rate, a total of 40 subjects will be recruited.

### 2.8 Recruitment

COPD patients receiving pharmacological treatment are recruited from the Division of Pulmonology of the Kyung Hee University Medical Center. This clinical trial is to be conducted at the Kyung Hee University Korean Medicine Hospital Clinical Trial Center. The purpose of this study is explained to the subjects, and screening is conducted among those who signed the informed consent form.

### 2.9 Data management and monitoring

Data are managed by independent third parties. All documents related to the clinical study will be safely stored by the principal investigator or the person in charge or head of the testing agency and the clinical study manager for data security. The source document for this study will be stored in a separate locked space or in a computer database with limited access. Only the principal investigator and sub-investigators delegated by the principal investigator can access the data. Monitoring may be carried out via phone or a regular visit to the testing agency for the clinical study. The monitor upon visit should check the original copy of the basic data of the subjects, record of procedures, and data storage (clinical study files). The monitor should also oversee the progress of the clinical study and discuss it with the investigator if a problem arises.

### 2.10 Ethics

This trial was approved by the IRB of the Kyung Hee University Korean Medicine Hospital (KOMCIRB 2020-12-006-002). The protocol complied with both the Declaration of Helsinki and the GCP Guidelines. Signed informed consent forms will be obtained from all eligible participants before enrollment.

This trial is registered with the Korean Clinical Trial Registry (KCT0006119).

### 2.11 Statistical methods

Statistical significance is based on the significance level 0.05 with a power of 0.8.

- Intended to treat, ITT: The subjects who underwent CMT at least once and performed the 6-MWT as the primary outcome.
- Per protocol, PP: The subjects who received the CMT intervention up to 80% or higher and performed the relevant tests.
- For all analyses, the ITT is the main analysis, while the PP is carried out simultaneously, and their results are presented together.

For continuous variables, the results are presented as mean and standard deviation, for which an independent *t* test or Mann–Whitney *U* test will be carried out. If the normality assumption is satisfied, an independent *t* test will be carried out; otherwise, the Mann–Whitney *U* test will be carried out. To adjust for the influence of age, ANCOVA with age as a covariate will be carried out. If a significant intergroup difference is found related to the baseline values, ANCOVA with age and baseline as covariates will be carried out. For missing values, the last measured value will be used for each subject based on the last observation carried forward (LOCF).

## 3 Discussion

The present protocol is the first attempt in South Korea to verify the effectiveness and safety of CMT for COPD. The effects of respiration rehabilitation are widely known, and exercise therapy is actively applied in clinical practice for the management of functional abilities in COPD patients.^[1]^ Exercise therapy is the optimal management therapy among non-pharmacological therapies, and it plays a role in improving the quality of life of COPD patients by improving symptoms such as dyspnea.^[29]^ However, voluntary participation in exercise therapy is not easy for patients with severe breathing difficulties. Therefore, the interest in MT performed by experts is increasing, and studies on COPD with MT have been conducted.^[14–18]^ MT plays a role in expanding the rib cage and reducing respiratory muscle shortening.^[30]^ Studies have shown a major effect on the improvement of motor performance in patients with COPD.^[16]^ The CMT used in this study is a technique involving 10 to 15 minutes of exercise conducted by a trained professional Korean medicine doctor, and as the patient can receive the therapy while lying on the bed, the technique is advantageous in that it can be performed regardless of the severity of the patient.

The results of randomized controlled trial (RCT) based on this protocol are anticipated to contribute to promoting the research on CMT in COPD treatment as well as being the starting point in establishing the clinical basis. Furthermore, the addition of an effective treatment for COPD, a chronic, intractable disease, will prove useful for clinicians and in the management of COPD patients.

### 3.1 Trial status

Currently, participant recruitment is ongoing. A full-scale, multicenter RCT is expected to be initiated in 2023 after analysis of the RCT data.

## Author contributions

**Conceptualization:** Hee-Jae Jung.

**Data curation:** Hee-Jae Jung, Kwan-Il Kim.

**Investigation:** Kwan-Il Kim, Beom-Joon Lee.

**Methodology:** Kwan-Il Kim.

**Project administration:** Kwan-Il Kim.

**Supervision:** Hee-Jae Jung, Beom-Joon Lee.

**Writing – original draft:** Kwan-Il Kim.

**Writing – review & editing:** Kwan-Il Kim, Beom-Joon Lee.
